# Influencing factors of fertilization failure during *in vitro* fertilization

**DOI:** 10.3389/fendo.2026.1880151

**Published:** 2026-08-07

**Authors:** Hongzhi Shi, Jiajia Liu, Rongrong Liu, Chen Li, Qi Song

**Affiliations:** 1 Department of Reproductive Medicine, Maternity and Child Care Center of Qinhuangdao, Qinhuangdao, Hebei, China; 2 Children Protect Branch, Maternity and Child Care Center of Qinhuangdao, Qinhuangdao, Hebei, China

**Keywords:** fertilization failure, *in vitro* fertilization, predictive modeling, risk factors, sperm morphology

## Abstract

**Objective:**

To identify independent risk factors for fertilization failure (FF) during conventional *in vitro* fertilization (IVF) and to develop a predictive model for clinical risk stratification.

**Methods:**

This retrospective study included 2,474 IVF cycles (173 FF cycles with fertilization rate <30% and 2,301 normal fertilization cycles with rate ≥30%) at a single center (2018–2023). Univariate and multivariate logistic regression analyses were performed to screen independent risk factors for FF. A combined predictive model was constructed using logistic binary regression, and its discriminative ability was assessed by the area under the ROC curve (AUC).

**Results:**

Multivariate analysis identified four independent risk factors for FF: high proportion of primary infertility (OR = 2.19, 95% CI: 1.48–3.25), low proportion of tubal factor (OR = 0.60, 95% CI: 0.42–0.86), low normal sperm morphology rate (OR = 0.81, 95% CI: 0.73–0.91), and low sperm concentration after treatment (OR = 0.94, 95% CI: 0.91–0.97). The combined predictive model achieved an AUC of 74.6% (95% CI: 70.3%–78.9%), with an optimal cutoff of 0.185 (sensitivity 68.2%, specificity 71.5%).

**Conclusion:**

Primary infertility, absence of tubal factor, low normal sperm morphology, and low post-treatment sperm concentration are independent predictors of FF. This combined prediction model shows moderate discriminative power for fertilization failure risk stratification. However, its low positive predictive value (15.1%) restricts accurate identification of high-risk IVF patients. The model can only be used as an auxiliary counseling tool rather than a definitive reference for choosing conventional IVF or ICSI; prospective multicenter external validation is required before clinical application.

## Introduction

Complete FF (fertilization rate =0) and low fertilization rate (fertilization rate <30%) during *in vitro* fertilization (IVF) treatment are collectively referred to as FF, and their incidence is about 5-20% (1, 2). FF is associated with a significant reduction in oocyte utilization and even no available embryos, and the early treatment cost, time spent, and mental and emotional dedication are irreparable, which is a shattering blow to doctors and patients.

Most of the studies on FF focus on male sperm parameters, and it has been found that the FF rate increases significantly with the decrease in sperm parameters (3). However, FF often occurs in patients with normal sperm parameters, and normal fertilization is observed in most patients with decreased sperm parameters in clinical practice, suggesting the presence of other factors affecting FF. Therefore, in addition to male sperm parameters, the whole treatment cycle of IVF, including female factors, ovulation induction (OI) and laboratory fertilization, was analyzed in the present study to discover independent risk factors for FF in these processes and construct prediction curves based on these independent influencing factors to provide references for the fertilization method selection and reduce the incidence of FF.

## Materials and methods

### Study subjects and grouping

Instead of the NF rate (4), a total FF of 30% was used as the cutoff point in the present study, considering that even abnormal fertilization also suggests the presence of fertilization ability of sperms and eggs, and the occurrence of abnormal fertilization is mainly related to the level of ovulation stimulating hormones (5) and the laboratory fertilization process (6).

Clinical data of IVF-assisted pregnancy(excluding ICSI)in Maternity & Child Care Center of Qinhuangdao between January 2018 and December 2023 were retrospectively analyzed. A total of 2,474 cycles were included and divided according to the fertilization rate. The enrolled cycles were grouped based on fertilization rate. A total of 75 cycles presented total fertilization failure with a fertilization rate of 0, and 98 cycles were defined as partial fertilization failure with a fertilization rate < 30%. According to the 2024 Chinese Expert Consensus on Conventional *In Vitro* Fertilization, total and partial fertilization failure were collectively termed fertilization failure. The normal fertilization group included 2301 cycles with a fertilization rate ≥ 30%.Because some patients in our center used short-term fertilization combined with rescue intracytoplasmic sperm injection (RICSI), FF in the RICSI cycles was determined based on the IVF fertilization rate before the RICSI procedure, and cycles with an IVF fertilization rate of <30% were included in the FF group. Basic data, OI and laboratory fertilization were compared between the two groups. Multivariate logistic regression analysis was performed with FF in IVF as the dependent variable and the indicators with statistically significant differences in the univariate analysis as independent variables to screen independent risk factors for FF in IVF. A ROC curve prediction model for FF using combined indicators was constructed by logistic binary regression fitting.

Inclusion criteria: females with an age of ≤42 years old and a number of retrieved oocytes of ≥3 at the first oocyte retrieval cycle.

Exclusion criteria: Patients with chromosomal abnormalities in either male or female partners, a history of previous ovarian surgery, severe systemic diseases that may affect the outcomes of controlled ovarian hyperstimulation (e.g., autoimmune diseases, hepatic and renal insufficiency), or abnormal oocyte maturity (MII oocyte rate ≤ 50%) were excluded.

### Methods

OI and egg retrieval An appropriate OI program was developed according to the age, ovarian reserve and other conditions of the patients.

Patients undergoing the long follicular phase protocol received a subcutaneous injection of 3.75 mg long-acting gonadotropin-releasing hormone agonist (GnRH-a) (Triptorelin Acetate for Injection, Decapeptyl, 3.75 mg/vial, Ipsen, France) on days 2 to 3 of the menstrual cycle. Once the pituitary down-regulation criteria (basal estradiol ≤ 50 pg/mL, basal luteinizing hormone ≤ 5 mIU/mL and basal follicle-stimulating hormone ≤ 5 mIU/mL) were met, controlled ovarian hyperstimulation was initiated with gonadotropins (Gn). For the long luteal phase protocol, one-third vial of short-acting GnRH-a (Triptorelin Acetate for Injection, Decapeptyl, 3.75 mg/vial, Ipsen, France) was subcutaneously administered during the mid-luteal phase, followed by Gn stimulation after satisfactory down-regulation. In the GnRH antagonist protocol, Gn was started on days 2 to 3 of the menstrual cycle at a routine daily dose of 150–300 IU. Five to six days later, when the dominant follicle reached 12–13 mm in diameter, 0.25 mg per day of GnRH antagonist (Cetrorelix Acetate Injection/Cetrotide, Merck, Germany or Ganirelix Acetate Injection/Orgalutran, Merck & Co., USA, 0.25 mg/vial for both) was added until the trigger day. The applied Gn preparations included Follitropin Alfa Injection (Gonal-f, 75 IU/vial, Merck, Germany), Follitropin Beta Injection (Puregon, 600 IU/vial, Merck & Co., USA) and Urofollitropin for Injection (Lishenbao, 75 IU/vial, Livzon Group, China), and the Gn dosage was adjusted according to serum hormone levels and follicular development during stimulation. When transvaginal ultrasound showed 2 to 3 dominant follicles with an average diameter of ≥ 18 mm in bilateral ovaries, one vial of Recombinant Human Chorionic Gonadotropin (Ovidrel, 250 μg/vial, Merck, Germany) was injected intramuscularly. Oocyte retrieval was performed 36–38 hours after triggering for long protocols and 34–36 hours for the antagonist protocol.

Male partners were required to abstain from ejaculation for 2–7 days. Semen samples were collected via masturbation and processed at room temperature (24–26 °C) after liquefaction. Sperm preparation was performed using density gradient centrifugation (SAGE, USA) combined with the swim-up method. Briefly, 1ml 80% high gradient solution was added to the bottom of a 15ml conical centrifuge tube, and 1ml 40% low gradient solution was gently placed on the top. The liquefied semen was gently added to the top layer and centrifuged at 100g for 20 mins. The supernatant was removed, and the precipitate was transferred to a new centrifuge tube containing 3ml fertilization medium (SAGE, USA), mixed well, and centrifuged at 300g for 5 mins. The supernatant was removed, and the precipitate was mixed and added to the bottom of a 5ml round-bottom centrifuge tube for swim-up. The volume of the swim-up solution in the round-bottom centrifuge tube was adjusted according to the amount of recovered precipitate, and 0.5ml of fertilization solution was usually used as the swim-up solution. The mixture was incubated in an incubator with 6% CO_2_ at 37 °C.

Fertilization methods (1) Long-term fertilization: 39-40hrs after hCG injection, the cumulus-oocyte complex was mixed with sperms and fertilized in a center well culture dish. The final fertilization concentration was adjusted to 100,000-300,000 sperms/ml according to the number of eggs in the dish, with no more than 12 eggs per dish. The granulosa cells were removed after 18–20 hours of culture, and the fertilization of oocytes was observed. (2) Short-term fertilization combined with RICSI: 39 -40hrs after hCG injection, the cumulus-oocyte complex was mixed with sperms. The granulosa cells were removed 5 hrs after fertilization, and the second polar body was observed in a new dish. If the ratio of oocytes with second polar body to mature oocytes (2pb rate) was >30%, no intervention was needed. If the 2pb rate was <30%, observation was performed again 1hr later. If the 2pb rate increased to >30%, no intervention was needed, and if the rate remained <30%, RICSI was performed on the oocytes with one polar body.

Determination of fertilization and calculation of fertilization rate The pronucleus was observed 18-20hrs after hCG injection. The number of fertilized eggs was calculated as the sum of 2PN + 1PN + multiple PN + 0PN, and the fertilization rate was calculated as the number of fertilized eggs/total eggs.

### Outcome measures

(1) Univariate analysis of FF during IVF. (2) Multivariate logistic regression analysis of FF during IVF. Factors with statistically significant differences in univariate analysis were included in the multivariate logistic regression analysis model to screen the independent risk factors for FF in IVF. (3) Logistic binary regression fitting was used to construct a ROC curve model of combined indicators to predict FF, and the area under the curve (AUC) was calculated.

### Statistical analysis

SPSS23.0 software was used for statistical analysis. The normality of continuous variables was assessed using the Shapiro-Wilk test, and homogeneity of variance was assessed using Levene’s test. As all continuous variables did not follow a normal distribution (Shapiro-Wilk test, P < 0.05), measurement data were presented as median [interquartile range, IQR], and the Mann-Whitney U test was used for comparison between groups. Enumeration data were presented as percentages, and the chi-square test was used for comparison between groups. Risk factors were screened using multivariate logistic regression analysis. Differences with a p value of <0.05 were considered statistically significant. Multicollinearity among independent variables was assessed using variance inflation factor (VIF) and tolerance values. A VIF < 5 and tolerance > 0.1 were considered indicative of no serious multicollinearity. Bootstrap internal validation with 1000 resamples with replacement was performed to quantify overoptimism of the predictive model. The optimism-corrected AUC was calculated to offset the overestimated discriminative performance caused by training and testing on the identical cohort.

## Results

Univariate analysis showed no significant differences in male age, female BMI, basal FSH, basal LH, basal PROL, AMH, blood glucose, TSH, FT4, Gn days, total Gn, concentrations of E2, LH, and P on hCG day, total number of eggs, and sperm concentration/egg (×10^4^ sperms/egg) between the FF group and the NF group (P>0.05 for all comparisons). However, there were significant differences in female age, infertile duration, initial dose of Gn, Gn dose/egg, sperm concentration before treatment, sperm motility, percentage of normal sperm morphology, sperm concentration after treatment, and fertilization concentration (10,000 sperms/ml), and all differences were statistically significant (all P<0.05) (**Table 1**).

**Table 1A:** Baseline patient characteristics of the FF and NF groups.

Items	The FF group (n=173)	The NF group (n=2301)	P
Female age(years), median (IQR)	31.00 (29.00-34.00)	32.00 (29.00-35.00)	0.010
Male age(years), median (IQR)	31.00 (29.00-35.00)	32.00 (30.00-35.00)	0.082
Infertile duration(years), median (IQR)	4.00 (3.00-5.00)	3.00 (2.00-5.00)	0.000
Female BMI(kg/m^2^), median (IQR)	23.60 (21.48-27.10)	23.80 (21.38-27.10)	0.791
Blood glucose	5.40 (5.18-5.63)	5.45 (5.15-5.72)	0.165
Primary infertility, n (%)	119(68.79%)	991(43.07%)*	0.000
Insulin resistance, n (%)	2(1.16)	41(1.78%)	0.760
PCOS, n (%)	38(21.97%)	475(20.64%)	0.752
Endometriosis, n (%)	9(5.20%)	90(3.91%)	0.526
Hypothyroidism, n (%)	6(3.47%)	83(3.61%)	1.000
Tubal factors, n (%)	85(49.13)	1614(70.14%)*	0.000

All continuous variables are presented as median (interquartile range, IQR) as they did not follow a normal distribution (Shapiro–Wilk test, P < 0.05). Categorical variables are presented as n (%). P values were calculated using the Mann–Whitney U test for continuous variables and the chi-square test for categorical variables.

**Table 1B:** Ovarian stimulation and laboratory parameters of the FF and NF groups.

Items	The FF group (n=173)	The NF group (n=2301)	P
Basal FSH(mIU/mL), median (IQR)	5.75 (4.72-6.83)	5.90 (5.01-7.06)	0.081
Basal LH(mIU/mL), median (IQR)	5.31 (3.94-7.25)	5.11 (3.65-7.47)	0.551
Basal PROL(mIU/L), median (IQR)	338.30 (252.40-464.55)	322.40 (239.10-435.40)	0.156
AMH(ng/mL), median (IQR)	3.45 (2.16-5.08)	2.96 (1.67-5.08)	0.018
Blood glucose(mmol/L), median (IQR)	5.40 (5.18-5.63)	5.45 (5.15-5.72)	0.165
TSH(mIU/L), median (IQR)	2.12 (1.50-2.78)	1.98 (1.47-2.68)	0.301
FT4(pmol/L), median (IQR)	16.10 (14.50-17.30)	16.10 (14.70-17.60)	0.235
Initial dose of Gn(IU), median (IQR)	225.00 (150.00-300.00)	225.00 (200.00-300.00	0.008
Gn days, median (IQR)	12.00 (10.00-13.00)	11.00 (10.00-13.00)	0.368
Total Gn dose(IU), median (IQR)	2625.00 (2175.00-3150.00)	2700.00 (2100.00-3400.00)	0.203
Gn dose/egg(IU/egg), median (IQR)	187.50 (135.00-300.00)	220.00 (129.41-380.00)	0.065
E2 on hCG day(pg/mL), median (IQR)	11010.00 (8772.00-17305.50)	11010.00 (7988.00-18965.25)	0.757
LH on hCG day(mIU/mL), median (IQR)	1.29 (0.74-2.81)	1.54 (0.90-2.87)	0.027
P on hCG day(ng/mL), median (IQR)	2.73 (1.86-4.25)	2.81 (1.78-4.19)	0.667
Total number of eggs, median (IQR)	14.00 (9.00-18.00)	13.00 (8.00-18.00)	0.120
Sperm concentration before treatment (×106/mL)	46.77 (24.05-77.83)	65.19 (40.73-107.34)	<0.001
Sperm motility before treatment (%), median (IQR)	26.00 (17.11-38.00)	35.65 (22.89-48.00)	0.000
Normal sperm morphology(%), median (IQR)	4.00 (3.00-5.00)	4.00 (4.00-6.00)	0.000
Sperm concentration after treatment (×106/mL)	8.00 (5.00-12.00)	10.00 (8.00-15.00)	0.000
Fertilization concentration(×104 sperm/mL)	24.00 (20.00-25.00)	24.00 (20.00-24.00)	0.015
Sperm concentration/egg(×104 sperm/egg)	1.71 (1.26-2.50)	1.82 (1.30-2.67)	0.307

All continuous variables are presented as median (IQR). P values were calculated using the Mann–Whitney U test. PROL, prolactin; AMH, anti-Müllerian hormone; TSH, thyroid-stimulating hormone; FT4, free thyroxine; Gn, gonadotropin; hCG, human chorionic gonadotropin.

Multivariate logistic regression analysis showed that a high percentage of primary infertility, a low percentage of tubal factors, a lower percentage of normal sperm morphology (OR = 0.811, 95% CI: 0.725-0.908, P<0.001), and lower sperm concentration after treatment (OR = 0.939, 95% CI: 0.906-0.973, P = 0.001) were independent risk factors for FF, and the differences were statistically significant (all P<0.05) (**Table 2**). Multicollinearity diagnostics were performed to assess the stability of the multivariate logistic regression model. As shown in **Table 3**, all VIF values ranged from 1.04 to 1.60, and all tolerance values exceeded 0.1, indicating no serious multicollinearity among the independent variables. This confirms the stability and reliability of the regression coefficients.

**Table 2:** Multivariate logistic regression analysis.

Items	B	SE	Wald	Significance	OR (95% CI)
Female age	0.028	0.025	1.299	0.254	1.028(0.979–1.080)
Infertile duration(years)	-0.061	0.032	3.607	0.058	0.941(0.884–1.002)
Presence of primary infertility	0.785	0.201	15.296	0.000	2.192(1.479–3.251)
Presence of tubal factors	-0.508	0.182	7.735	0.005	0.602(0.421–0.860)
Gn dose/egg	0.001	0.001	1.055	0.304	1.001(0.999–1.003)
Initial dose of Gn	0.001	0.002	0.227	0.634	1.001(0.997–1.005)
Sperm concentration	0.002	0.002	1.042	0.307	1.002(0.998–1.006)
Sperm motility	0.009	0.006	2.554	0.110	1.009(0.997–1.021)
Percentage of normal sperm morphology	-0.209	0.057	13.154	0.000	0.811(0.726–0.907)
Sperm concentration after treatment	-0.063	0.018	11.587	0.001	0.939(0.906–0.973)
Fertilization concentration	-0.013	0.029	0.214	0.643	0.987(0.933–1.045)
Constant	-0.292	1.052	0.077	0.781	0.747(0.095–5.870)

Statistically significant (P < 0.05 and 95% CI excludes 1.0).

**Table 3:** Multicollinearity diagnostics for the multivariate logistic regression model.

Variable	VIF	Tolerance	Assessment
Female age	1.291	0.774	No collinearity
Infertile duration	1.102	0.907	No collinearity
Presence of primary infertility	1.184	0.845	No collinearity
Presence of tubal factors	1.076	0.929	No collinearity
Gn dose/egg	1.598	0.626	No collinearity
Initial dose of Gn	1.48	0.675	No collinearity
Sperm concentration before treatment	1.46	0.685	No collinearity
Sperm motility before treatment	1.037	0.965	No collinearity
Percentage of normal sperm morphology	1.077	0.929	No collinearity
Sperm concentration after treatment	1.432	0.698	No collinearity
Fertilization concentration	1.219	0.821	No collinearity

VIF < 5 indicates no serious multicollinearity; tolerance > 0.1 is acceptable.

**Figure 1:**
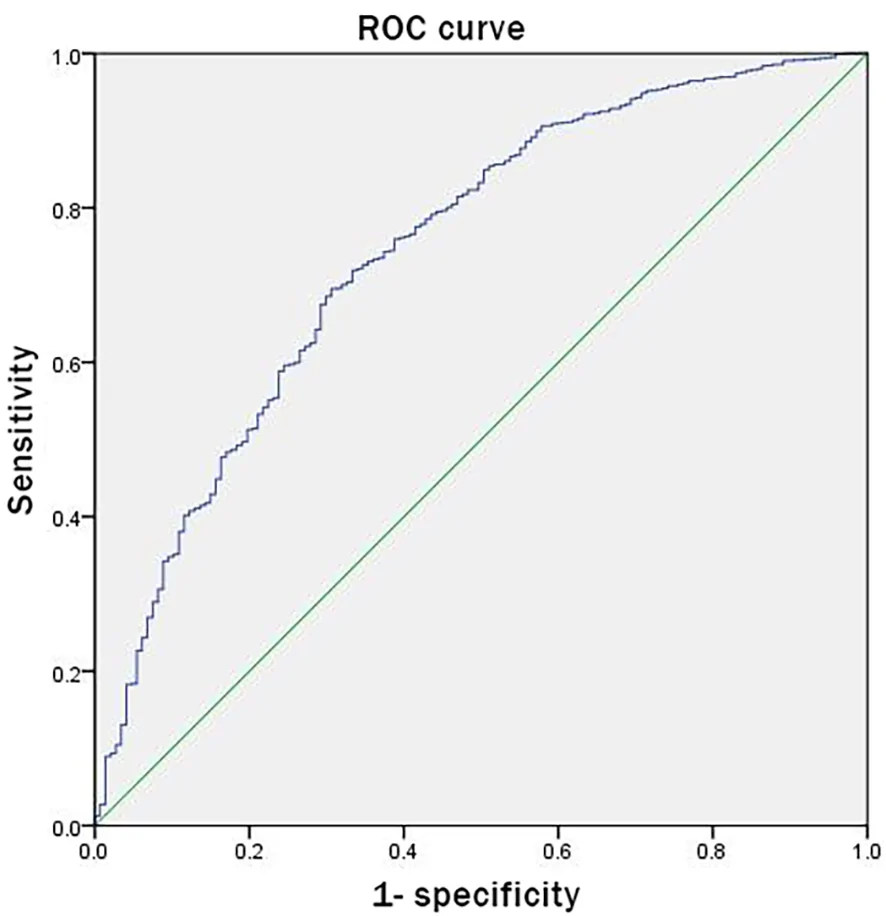
ROC curve for predicting fertilization failure using combined indicators. The model achieved an AUC of 74.6% (95% CI: 70.3%–78.9%). At the optimal cutoff value of 0.185 (determined by the Youden index), the sensitivity was 68.2%, specificity was 71.5%, PPV was 15.1%, and NPV was 96.9% (assuming a 7% prevalence of fertilization failure).

**Figure 2:**
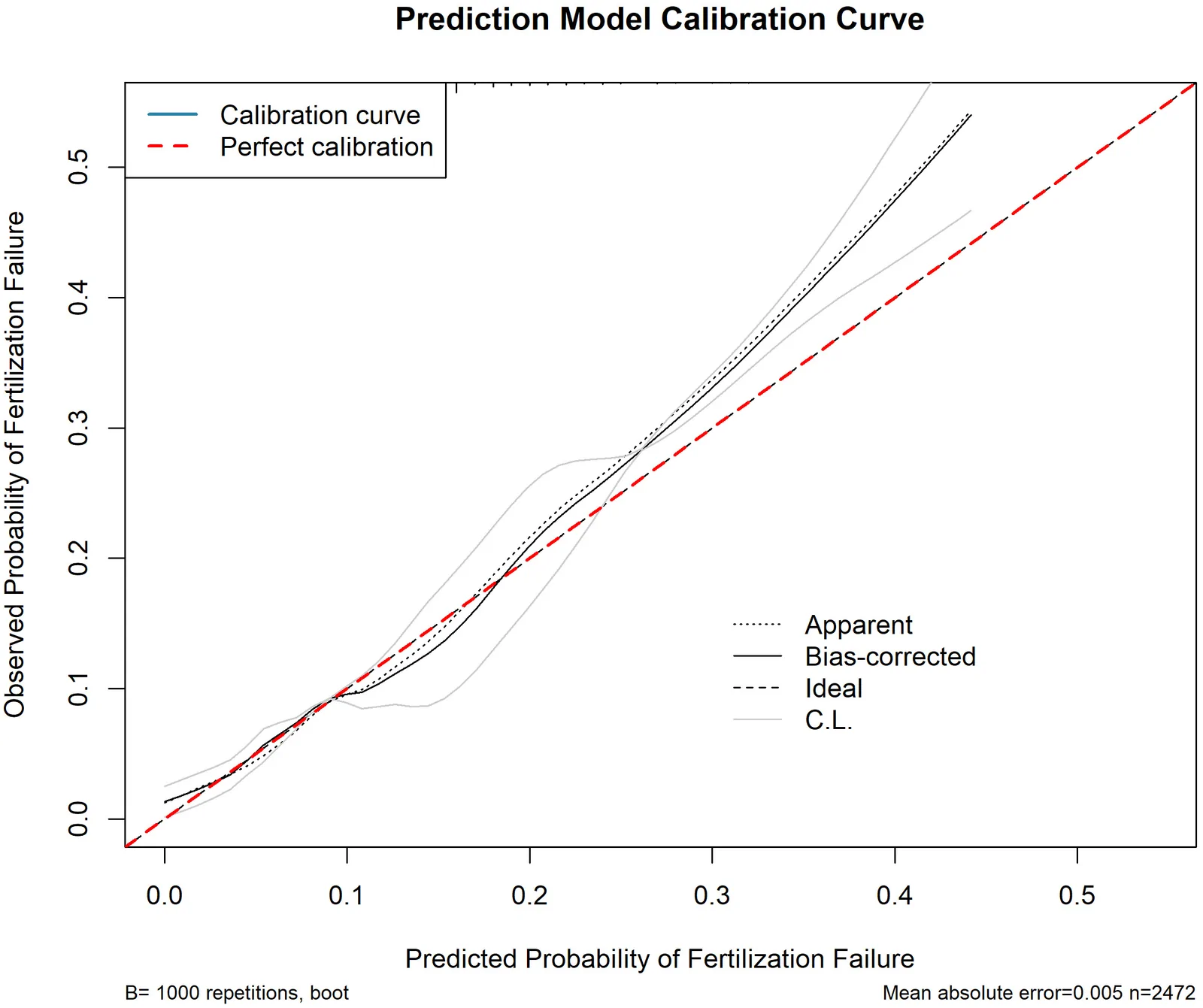
Calibration curve for the combined IVF fertilization failure prediction model, with Hosmer-Lemeshow χ^2^=8.62 (P=0.375) and Brier score of 0.19.

Logistic binary regression fitting was used to construct a ROC curve prediction model for the combined prediction of FF using various indicators, and the AUC was 74.6% (**Figure 1**). The combined prediction model yielded an apparent AUC of 0.746 (95% CI: 0.703–0.789). After 1000 bootstrap resampling internal validation, the optimism-corrected AUC was 0.712, confirming mild overoptimism of the uncorrected value derived from a single dataset. Model calibration was evaluated via the Hosmer-Lemeshow goodness-of-fit test and Brier score. The Hosmer-Lemeshow test showed χ^2^ = 8.62, df = 8, P = 0.375, indicating no statistically significant difference between predicted and observed fertilization failure risks, demonstrating favorable model fit. The Brier score of the model was 0.19, further verifying consistent predicted and actual event probabilities. A calibration plot was generated to visually display model calibration performance (**Figure 2**). Using the Youden index, the optimal cutoff value for the combined prediction model was determined to be 0.185, corresponding to a sensitivity of 68.2% and a specificity of 71.5%. At this cutoff, the positive predictive value (PPV) was 15.1% and the negative predictive value (NPV) was 96.9%, given a 7% prevalence of FF in the study population. The high NPV (96.9%) indicates that the model is particularly useful for ruling out FF in low-risk patients.

## Discussion

A total of 2,474 cycles were included in this study, of which 173 cycles were included in the FF group(75 cycles with complete FF and 98 cycles with partial FF), with a FF rate of 7%, which was slightly lower than that reported previously (1). This was possibly explained by the fact that only cycles with a number of eggs ≥3 and a female age ≤42 years old were selected, and cycles with abnormal egg maturation were excluded.

In patient factor analysis, in addition to general factors such as age, infertile duration, BMI, ovarian function parameters, and presence of primary infertility, the endocrine factors (7) that may affect egg quality were also analyzed, including blood glucose related to islet function (8), thyroid function (9), polycystic ovary syndrome(PCOS) (10), and endometriosis (11), and no differences were observed between the two groups. It is notable that age and infertile duration were not independent influencing factors of FF in the present study.

The process of OI affects the quality of eggs (12). Comparisons of the parameters such as Gn dose, Gn days, concentration of E2, LH, and P on hCG day, and total number of eggs revealed no differences. Factors with significant differences in univariate analysis, including the initial dose of Gn and Gn dose/egg, were not independent influencing factors in logistic regression analysis. Due to the complexity of the OI process, many individualized procedures are involved. Different study and grouping methods may lead to different conclusions. The absence of differences did not necessarily indicate that the OI process has no effect on fertilization, and future studies on this topic are needed.

Although significant differences in sperm concentration before treatment and sperm motility were noted in univariate analysis, they were not independent influencing factors of FF in logistic binary regression analysis, which may be explained by the fact that although the quality of sperm from each male was different, the final fertilization concentration can be adjusted in laboratory by increasing the amount of sperm to control the final fertilization concentration (10,000 sperms/egg, 10,000 sperms/ml) in a certain range (13).

In addition, there were differences in different semen analysis software and analysis methods (14), and unified external quality control was absent. The present study showed that the percentage of normal sperm morphology is an independent influencing factor of FF, which was consistent with the findings of other studies (15). Therefore, the percentage of normal sperm morphology is of important value in the evaluation of male fertility.

In terms of laboratory factors, a difference in the sperm recovery rate is noted when different treatment methods and sperm separation solutions are used (16). Density gradient centrifugation combined with swim-up was used in our hospital. Semen samples with different qualities were treated using the optimal treatment method in 5ml round bottom centrifuge tube with 0.5 ml culture medium, and no difference in sperm motility was found after treatment. Furthermore, the selection of fertilization methods is basically based on the quality of sperm processed on the same day. Therefore, the concentration of motile sperms in the swim-up solution is more representative of the total number of forward moving sperms, and can serve as an independent influencing factor of FF, which was consistent with the results of other studies (17).

In the selection of fertilization methods, due to the different sperm quality standards for implementing ICSI (17, 18), it is inevitable that changes in the percentage of ICSI may affect the incidence of FF in IVF because different sperm quality standards are used when ICSI procedure is performed, which is also one of the reasons for the significant difference in FF rates reported. In our center, the sperm quality standard for ICSI was a swim-up sperm concentration of ≤ 2x10^6^/ml, or a total number of motile sperm of ≤ 1x10^6^. If the cutoff value increases, the percentage of ICSI as well as the consequent risk will increase (19), and if the cutoff value decreases, the incidence of FF will increase.

In addition to the clinical characteristics of patients, complete FF may also be induced by genetic defects. NF can be achieved in some cycles through ICSI (20), and some cycles, however, require ICSI combined with artificial oocyte activation (21), which was not discussed in the present study.

Among patients with primary infertility, ovulatory dysfunction is the most common female factor. Since this study focused on conventional IVF cycles, female-related factors were not discussed in detail. Primary male infertility is predominantly associated with sperm quality, particularly normal sperm morphology rate. Abnormal spermatozoa impair fertilization through multiple mechanisms. Although conventional IVF facilitates sperm-oocyte contact, it still requires sperm to penetrate the oocyte spontaneously, a process dependent on the acrosome reaction. Sperm with head defects often present impaired acrosomal function and reduced DNA integrity. Abnormalities in the midpiece and tail can compromise sperm motility, ultimately leading to poor fertilization outcomes.

Our findings confirm that low normal sperm morphology rate is an independent predictor of FF, consistent with previous reports (15). Morphologically abnormal sperm often exhibit impaired acrosomal function and reduced DNA integrity, compromising zona pellucida penetration (13). Primary infertility was the strongest risk factor (OR = 2.19), possibly reflecting underlying gamete interaction defects, whereas tubal factor was protective (OR = 0.60), indicating preserved gamete function. Notably, female age and infertile duration were not independent predictors after multivariable adjustment, suggesting their effects may be mediated through other factors such as ovarian reserve.

The ROC-based prediction model developed in this study has potential clinical utility in several aspects. First, the model can assist clinicians in identifying patients at high risk of FF prior to the IVF cycle. For example, patients with a predicted probability exceeding the optimal cutoff (0.185) may be counseled regarding their increased risk of FF. Second, the model can guide the selection of fertilization strategies. In high-risk patients, clinicians may consider proceeding directly with intracytoplasmic sperm injection (ICSI) rather than conventional IVF, or implementing a short-term fertilization protocol combined with rescue ICSI (RICSI). Third, the model facilitates evidence-based patient counseling. Clinicians can use the predicted probability to inform patients about their individualized chance of successful fertilization, thereby managing expectations and supporting shared decision-making. Clinicians can use the predicted probability to inform patients about their individualized chance of successful fertilization, thereby managing expectations and supporting shared decision-making. Recently, Wang et al. (22) developed separate nomogram models for LFR and TFF using a large cohort of 11,598 patients, achieving AUCs of 0.640 and 0.899, respectively. Their TFF model incorporated LH and progesterone levels on hCG day and number of oocytes retrieved, which partially overlap with our findings regarding female factors. However, their model did not include post-treatment sperm concentration or normal sperm morphology as independent predictors, which we identified as critical male factors in our study. Furthermore, Liu et al. (23) constructed a nomogram for FF prediction (AUC = 0.776) using factors including infertility duration, sperm concentration, and basal estrogen levels. While their model demonstrated moderate predictive ability, it did not differentiate between primary and secondary infertility as a risk factor, which was the strongest predictor in our model (OR = 2.19). These comparisons highlight that our model extends previous work by integrating both post-treatment sperm parameters and infertility etiology into a unified risk stratification tool. The model’s positive predictive value was only 15.1%, meaning most patients predicted as high risk would not develop fertilization failure, which limits its capacity to screen candidates requiring early ICSI intervention. By contrast, the model had an excellent negative predictive value of 96.9%, which is valuable for reassuring low-risk patients and avoiding unnecessary ICSI. We caution that this model cannot independently guide clinical decision-making for fertilization modality selection; it merely provides supplementary risk information for patient communication pending external validation.

Limitations and prospects: Several limitations of this study should be acknowledged. First, this was a single-center retrospective study with only internal bootstrap validation performed; no external prospective multicenter validation was conducted. The model’s discriminative performance may still be overestimated, and its generalizability to other reproductive centers requires further verification. The retrospective design inherently carries the risk of selection bias and unmeasured confounding. Although we applied strict inclusion and exclusion criteria, the possibility of residual confounding cannot be excluded. Second, the number of FF cycles was relatively small (n = 173), which may limit the statistical power and stability of the logistic regression model. With only 173 events, the model included 11 independent variables, which approaches the recommended limit of 10–15 events per variable. Therefore, overfitting is a potential concern. Third, the study population was derived from a single reproductive center with specific patient demographics and treatment protocols. The FF rate in our center was 7%, which is slightly lower than that reported in some other studies. Consequently, the generalizability of our findings to other populations or centers with different characteristics may be limited. Fourth, although we identified several independent risk factors for FF, the AUC of the combined prediction model was 74.6%, indicating only moderate discriminative ability. This suggests that additional, yet-unmeasured factors—such as genetic defects (e.g., acrosin deficiency, PLCζ mutations) or sperm functional abnormalities—may also contribute to FF. These factors were not assessed in the present study. Fifth, semen parameters were analyzed using routine laboratory methods rather than standardized external quality control protocols. Variations in semen analysis software and techniques across centers may affect the reproducibility of our findings. Finally, the prediction model was developed without external validation. Internal validation techniques (e.g., bootstrap or cross-validation) were not applied due to the limited sample size. Future directions: Prospective, multicenter cohort studies with larger sample sizes are warranted to externally validate and refine the predictive model. Future research should also explore the incorporation of biomarkers—such as anti-Müllerian hormone (AMH) as reported by Vale-Fernandes et al. (24), genetic testing, and sperm functional assays—to improve the model’s predictive performance. Until such validation is completed, the model should be used as a decision-support tool rather than a definitive diagnostic test.

## Conclusion

Primary infertility, non-tubal infertility, low normal sperm morphology rate and low post-processing sperm concentration are independent risk factors for IVF fertilization failure. The combined prediction model has moderate discriminative performance but limited high-risk screening capacity due to low PPV. It can serve as a preliminary risk stratification tool for patient counseling, but cannot be routinely applied to select IVF/ICSI protocols until validated by multicenter prospective cohorts.

## Data Availability

The raw data supporting the conclusions of this article will be made available by the authors, without undue reservation.
